# Numerical Simulation of Laser Cladding Using Cable Wires

**DOI:** 10.3390/ma19112326

**Published:** 2026-06-01

**Authors:** Weihang Liu, Xueping Guo, Kaiyong Jiang, Jian Liu, Zhaoju Peng, Xizhao Lu, Jianming Zhang, Zhihai Cai, Dehua Wu, Yuchao Xu, Binggong Yan

**Affiliations:** 1 School of Shipping and Maritime Studies, Guangzhou Maritime University, Guangzhou 510725, China; 2 Fujian Key Laboratory of Special Energy Manufacturing, College of Mechanical Engineering and Automation, Huaqiao University, Xiamen 361021, Chinapengzhaojuhqu@163.com (Z.P.);; 3 National Engineering Research Center for Remanufacturing, PLA Army Services University, Beijing 100072, China

**Keywords:** laser cladding, high entropy alloys, cable-wire, simulation, comsol

## Abstract

Cable wires provide a viable technical pathway for the laser additive manufacturing of high-entropy alloys (HEAs). However, the complex interplay of structural and material parameters of cable wires leads to significant variations in molten pool dynamics, which poses challenges to the fabrication of high-quality HEA coatings. To clarify the effects of these key factors on molten pool behavior, a multi-physics numerical model for the laser cladding of Al_50_Si_6_Ti_8_Cr_12_Cu_12_Ni_12_ cable wires was established in this study. A dedicated physical model for cable wires was developed, and the Level Set Method was employed to track fluid interfaces throughout the cladding process. Based on the proposed model, the temperature distribution, stress fields, and elemental homogeneity within the molten pool were systematically investigated. The results reveal that chromium (Cr) addition induces a viscosity reduction, and a torsional pitch of ≤4 mm is critical for achieving defect-free, compositionally uniform HEA coatings, which provides novel insights for process optimization and alloy design of cable-wire laser cladding.

## 1. Introduction

High-entropy alloys (HEAs) have revolutionized conventional alloy design paradigms by departing from reliance on a single principal element. Exhibiting superior strength, hardness, wear resistance, and corrosion resistance, HEAs are regarded as promising candidates for advanced engineering applications [1,2]. Nevertheless, severe elemental segregation and poor room-temperature ductility pose substantial challenges to the fabrication of high-quality HEA wires, and no mature industrial manufacturing technology has been established to date. This bottleneck significantly impedes the development of wire-fed additive manufacturing for HEAs [3,4,5]. Cable wires have emerged as a feasible alternative, providing a viable pathway for the practical engineering implementation of HEA wire-fed additive manufacturing [6,7,8,9,10,11,12].

Compared with single-component wires, the multi-strand twisted structure of cable wires introduces greater complexity to the laser cladding process. During cladding, the interaction between the laser and the multi-metal strands intensifies laser reflection and absorption, enhances plasma impact, and strengthens Marangoni convection in the molten pool [13]. During solidification, it promotes more pronounced dendritic growth and residual stress accumulation. These phenomena make cable-wire laser cladding highly susceptible to defects such as porosity, cracking, and compositional segregation [14,15], which severely affect the quality and performance of HEA coatings. At present, research on cable-wire laser cladding mainly focuses on experimental exploration, while the theoretical analysis of its complex physical and chemical processes remains insufficient. The underlying mechanisms by which cable-wire structural/material parameters, laser process variables, and in situ molten pool metallurgical reactions jointly influence molten pool behavior, microstructure evolution, and coating performance are still unclear. This limits the further optimization of process parameters and the design of high-performance HEA cable wires.

To address the above research gaps, this study takes the self-designed Al_50_Si_6_Ti_8_Cr_12_Cu_12_Ni_12_ HEA cable wire as the research object [12]. Using the COMSOL Multiphysics 5.5 multi-physics simulation platform, a fully coupled numerical model of cable-wire laser cladding is established [16]. A dedicated physical model for the twisted multi-strand structure of cable wires is constructed, and the Level Set Method is adopted to accurately track the gas–liquid interface evolution during cladding. Through systematic simulation, the temperature field, flow field, stress field, and elemental distribution characteristics in the molten pool are quantitatively analyzed. The effects of key factors (including cable-wire torsional pitch, alloy composition, and laser power) on molten pool dynamics, solidification behavior, and coating quality are revealed. The research results aim to clarify the internal correlation between process parameters, molten pool behavior, and coating performance, providing theoretical guidance for the optimization of cable-wire laser cladding processes and the design of high-performance HEA coatings, and promoting the industrial application of HEA cable-wire laser cladding technology.

## 2. Experimental

### 2.1. Materials

**Figure 1:**
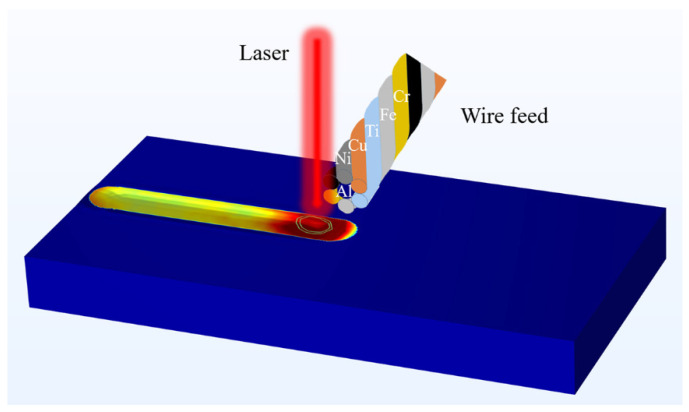
Schematic illustration of cable-wire laser cladding.

An in-house designed cable wire Al_50_Si_6_Ti_8_Cr_12_Cu_12_Ni_12_ was utilized in this study. The core strand consisted of a 0.8 mm diameter ER4047 Al-Si alloy wire, while the outer strands were composed of 0.3 mm diameter wires made of Ti, Cu, Ni, and Cr. For the substrate material, 45# steel substrates were selected. The laser cladding parameters, which had been previously optimized by the authors [12], were employed under the following conditions: laser power of 2000 W, scanning speed of 0.2 mm s^−1^, and wire-feed rate of 100 mm min^−1^. A schematic diagram of the experimental setup is provided in Figure 1.

### 2.2. Equipment

**Figure 2:**
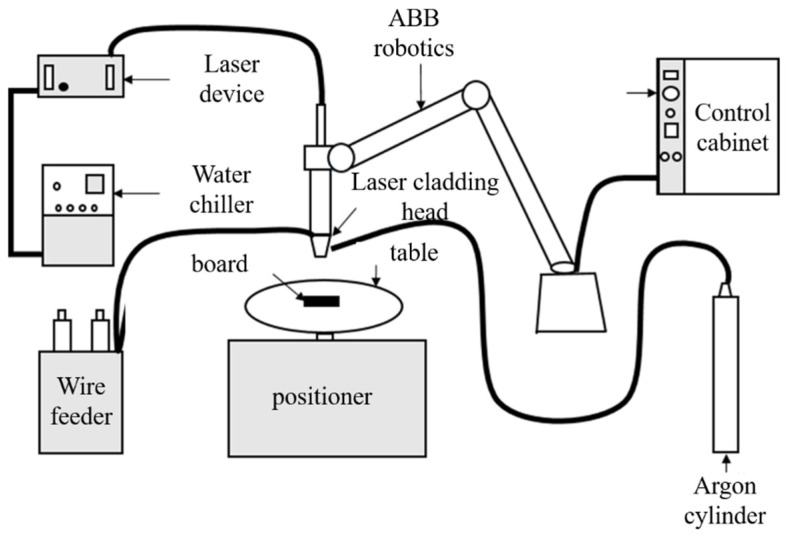
Experimental setup for laser cladding.

The experimental setup, as illustrated in Figure 2, consists of a 2500 W LDM-2500 direct-diode laser (LaserLine, Mülheim-Kärlich, Germany) with a 6 mm diameter top-hat intensity beam profile, an ABB IRB4600 six-axis industrial robotic system (Zurich, Switzerland), and high-purity argon gas (99.999%) used as a shielding gas, with a shielding gas pressure of 0.4 Mpa. The laser cladding nozzle performs a unidirectional scan at a position 10 mm away from the substrate.

## 3. Models

**Figure 3:**
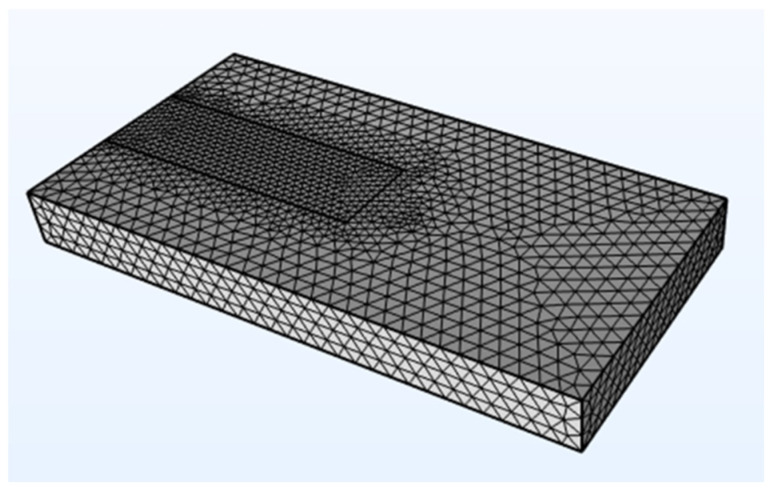
Computational mesh.

To investigate elemental mixing, melt-pool flow, solidification behavior, and stress distribution during cable-wire laser cladding, a multi-physics model of the melt-pool was developed using COMSOL Multiphysics 5.5 and executed on an Intel^®^ Core™ i7-7700 CPU (8 GB RAM, Windows 10 operating system). Effective thermophysical properties were calculated as volume-fraction-weighted averages of the constituent wire and molten substrate materials. The substrate dimensions were set to 50 mm × 100 mm × 10 mm. To ensure numerical convergence and model fidelity, the computational mesh was locally refined within the melt-pool and its surrounding regions (Figure 3). Due to the inherent complexity of the process, several simplifying assumptions were introduced [17,18,19,20]:

(1) The melt behaves as an incompressible, laminar Newtonian fluid.
(2) Materials are isotropic.
(3) The laser beam is an ideal top-hat source with negligible variation along z.
(4) Momentum transfer induced by wire feeding is neglected, as the wire feed rate (100 mm/min) is two orders of magnitude lower than the characteristic melt-pool flow velocity, rendering its contribution to the overall momentum balance insignificant.
(5) Vaporization of aluminum is neglected regarding its influence on the clad geometry.
(6) Temperature-dependent thermophysical data were taken from JMatPro 7.0.

### 3.1. Control Equations

The mass conservation equation [21,22] is:

(1) $$\frac{\partial \rho }{\partial t}+\nabla \left(\rho \cdot u\right)=0$$

The momentum conservation equation [21,22] is:

(2) $$\rho \frac{\partial u}{\partial t}+\rho \left(u\cdot \nabla \right)u=\nabla \cdot \left[-p\cdot I+\tau \right]+F$$

(3) $$F=p_{\mathrm{bg}}+p_{M}+p_{\mathrm{sat}}$$

Here, F denotes the body-force vector, with $p_{\mathrm{bg}}$ the buoyancy force, $p_{M}$ the Marangoni force, and $p_{\mathrm{sat}}$ the recoil-pressure force.

The conservation of thermal energy [21,22] is:

(4) $${\rho C}_{p}u\cdot \nabla T+\nabla \cdot q=Q_{\mathrm{las}}+Q_{\mathrm{pt}}+Q_{\mathrm{loss}}$$

q=−k∇T

Here, Q_las_ denotes the effective laser heat input, Q_pt_ the latent heat of phase change, and Q_loss_ the heat losses arising from convection and radiation.

The free surface of the melt-pool is tracked by the Level Set Method, in which the level-set variable φ represents the fluid volume fraction within each computational cell. Specifically, φ = 0 indicates that the cell is entirely filled by fluid 1 (gas), whereas φ = 1 signifies that the cell is completely occupied by fluid 2 (liquid). Conservation of the level-set variable is governed by:

(5) $$\frac{\partial \varphi }{\partial t}+u\cdot \nabla \varphi =\gamma \nabla \cdot \left(\epsilon_{\mathrm{ls}}\nabla \varphi -\varphi \left(1-\varphi \right)\frac{\nabla \varphi }{\left|\nabla \varphi \right|}\right)$$

Using the level-set variable φ, the volume-averaged and mass-averaged properties across the gas–liquid interface are computed as:

(6) $$\rho_{\mathrm{surface}}={\varphi \rho }_{l}+\left(1-\varphi \right)\rho_{g}$$

(7) $$c_{\mathrm{purface}}=\frac{{\varphi \rho }_{l}c_{\mathrm{pl}}+\left(1-\varphi \right)\rho_{g}c_{\mathrm{pg}}}{{\varphi \rho }_{l}+\left(1-\varphi \right)\rho_{g}}$$

where $\rho_{l}$ and $\rho_{g}$ denote the densities of the liquid and gas phases, respectively, while $c_{\mathrm{pl}}$ and $c_{\mathrm{pg}}$ represent their corresponding specific heat capacities.

### 3.2. Laser Heat Source Model

A top-hat laser beam was employed in the experiments. The heat flux density $q_{\mathrm{las}}$ is expressed as:

(8) $$q_{\mathrm{las}}=\frac{\eta_{\mathrm{las}}P_{\mathrm{las}}}{{{\pi r}_{\mathrm{las}}}^{2}}{*f}_{1}$$

(9) $$f_{1}=\left\{\begin{array}{c} 1\left(x^{2}+y^{2}\leq r_{\mathrm{las}}\right)\\ 0(x^{2}+y^{2}>r_{\mathrm{las}}) \end{array}\right.$$

where $f_{1}$ is a step function, $\eta_{\mathrm{las}}$ the effective laser-energy absorption coefficient, $P_{\mathrm{las}}$ the laser power, and $r_{\mathrm{las}}$ the beam-spot radius. During cladding, a fraction of the laser energy is reflected at the surface, another fraction is defocused by the shielding gas and plasma plume, and only a small portion is effectively absorbed. For the 1030 nm wavelength, $\eta_{\mathrm{las}}$ can be estimated as a function of surface temperature T [23]:

(10) $$\eta_{\mathrm{las}}=354.67\sqrt{10^{-8}\left(a_{\Omega }+b_{\Omega }T\right)}$$

with temperature-dependent electrical-resistivity constants: $a_{\Omega }$ = 22.1 and $b_{\Omega }$ = 0.11 for 273 K < T < 1150 K, and $a_{\Omega }$ = 148.6 and $b_{\Omega }$ = 0 for 1150 K < T < 1953 K.

### 3.3. Boundary

The total heat loss $q_{\mathrm{loss}}$ from the melt-pool comprises radiation $q_{\mathrm{rad}}$ and convection $q_{0}$:

Radiative heat loss [24]:

(11) $$q_{\mathrm{rad}}=-\varepsilon_{\mathrm{rad}}\sigma_{s}\left(T^{4}-{T_{\mathrm{ref}}}^{4}\right)$$

where ε_rad_ is the surface emissivity, σ_s_ the Stefan–Boltzmann constant, and T_ref_ the ambient temperature.

Convective heat loss [24]:

(12) $$q_{0}=-h_{A}\left(T-T_{\mathrm{ref}}\right)$$

with h_A_ the convective heat-transfer coefficient. During melting and solidification, latent heat is absorbed or released. The specific enthalpy change in the mushy zone is expressed as [24]:

(13) $$\Delta H_{\mathrm{pt}}=H_{\mathrm{ref}}+\overset{T}{\underset{T_{\mathrm{ref}}}{\int }}C_{p}\mathrm{dT}+\Delta H_{m}f_{l}$$

where H_ref_ denotes the reference enthalpy, ΔH_m_ the latent heat of fusion, and f_l_ the liquid volume fraction in the mushy zone. Consequently, the source term accounting for latent-heat exchange is:

(14) $$q_{\mathrm{pt}}=-\frac{\partial }{\partial t}\left(\rho \Delta H_{\mathrm{pt}}\right)-\nabla \left(\rho u\Delta H_{\mathrm{pt}}\right)$$

### 3.4. Surface and Body Forces in the Melt-Pool

During the laser cladding process, the pressure generated by the free expansion of metal vapor above the melt surface significantly exceeds atmospheric pressure and serves as a primary driving force for melt-pool flow. The recoil pressure P_sat_ exerted by the metal vapor can be expressed as follows [25]:

(15) $$P_{\mathrm{sat}}=0.54P_{0}\exp\left(\Delta H_{\mathrm{LV}}\frac{M\left(T-T_{a}\right)}{{\mathrm{RTT}}_{a}}\right)\vec{n}\frac{2\bar{\rho }}{\rho_{l}+\rho_{g}}$$

where ΔH_LV_ is the latent heat of vaporization, M the relative atomic mass, R the universal gas constant, and T_a_ the vaporization temperature.

Buoyancy arising from thermal expansion and density gradients within the molten metal opposes the gravitational force. The coupled buoyancy–gravity volumetric source term is given by [24]:

(16) $$P_{\mathrm{bg}}=\rho g-\rho \alpha \left(T-T_{m}\right)g$$

with α the thermal expansion coefficient and T_m_ the melting point.

The surface tension coefficient is temperature-dependent. Temperature gradients across the melt surface induce Marangoni convection. The corresponding momentum source is:

(17) $$P_{M}=\frac{d\gamma }{\mathrm{dT}}\left[\nabla T-\left(\vec{n}\nabla T\right)\vec{n}\right]\frac{2\bar{\rho }}{\rho_{l}+\rho_{g}}$$

The temperature-dependent surface tension γ is modeled as [5]:

(18) $$\gamma =\gamma_{m}^{0}-\beta_{\mathrm{st}}\left(T-T_{\mathrm{ref}}\right)-{\mathrm{RT}\Gamma }_{s}\ln\left[1+a_{i}k_{\mathrm{es}}\exp\left(-\frac{\Delta H^{0}}{R_{g}T}\right)\right]$$

where $\gamma_{m}^{0}$ is the surface tension at the reference temperature, β_st_ the temperature coefficient of surface tension, Γ_s_ the surface excess at saturation, ai the activity of the solute, k_es_ the entropy separation constant, and ΔH^0^ the segregation enthalpy.

### 3.5. Thermo-Mechanical Model for the Clad Track

The differential thermal expansion of the metal during laser cladding gives rise to transient thermal stresses, which evolve into residual stresses upon cooling. These residual stresses represent a primary cause of cracking [26,27]. Therefore, numerical simulation of the stress field during solidification serves as an effective approach for crack prediction and control [28,29,30]. A sequentially coupled thermo-mechanical analysis was conducted using COMSOL Multiphysics: the transient temperature field was first calculated and then applied as a thermal load to the solid mechanics module to determine the spatiotemporal evolution of the stress distribution.

Due to the highly non-uniform temperature distribution and cooling rate within the melt-pool, significant residual stresses were generated. The von Mises yield criterion was employed to determine the onset of plastic deformation [31,32,33]. In principal stress space, the equivalent stress is:

(19) $$\sigma =\frac{\sqrt{2}}{2}\sqrt{\left(\sigma_{1}-\sigma_{2}\right)+\left(\sigma_{2}-\sigma_{3}\right)+\left(\sigma_{3}-\sigma_{1}\right)}$$

where σ_1_, σ_2_, σ_3_ are the principal stresses.

The incremental plastic strain {dε}_p_ is related to the stress state through the flow rule [34]:

(20) $${\left\{d\varepsilon \right\}}_{p}=d\lambda \frac{\partial \bar{\sigma }}{\partial \left\{\sigma \right\}}$$

with dλ the plastic multiplier and $\frac{\partial \bar{\sigma }}{\partial \left\{\sigma \right\}}$ the gradient of the yield surface.

Hardening is described by [35]:

(21) $$\sigma_{y}=\sigma_{y0}+\sigma_{h}$$

where σy_0_ denotes the initial yield strength and σ_h_ the isotropic hardening function.

### 3.6. Mathematical Model of Clad-Track Geometry

The relationship between process parameters and the resulting clad geometry constitutes a fundamental basis for laser cladding design and analysis, and has therefore been extensively studied [36]. Current predictive methodologies can be broadly classified into two categories: (i) theoretical models based on cladding thermodynamics and idealized powder-flow fields [37,38,39,40], and (ii) empirical models derived from experimental observations [41,42]. The former necessarily rely on simplifying assumptions and are generally capable of providing only qualitative trend predictions with limited quantitative accuracy. In contrast, the latter have predominantly been developed for powder-fed systems and are seldom reported for wire-fed cladding processes—particularly in the context of cable-wire cladding.

In this study, an empirical mathematical model is proposed to predict the clad-layer geometry resulting from cable-wire laser cladding. The model establishes a functional relationship between process parameters and geometric characteristics, and is implemented within an Arbitrary Lagrangian–Eulerian (ALE) moving-mesh finite element framework to simulate the real-time accumulation of molten metal within the melt-pool.

#### 3.6.1. Derivation of Process-Parameter–Geometry Relationships

**Figure 4:**
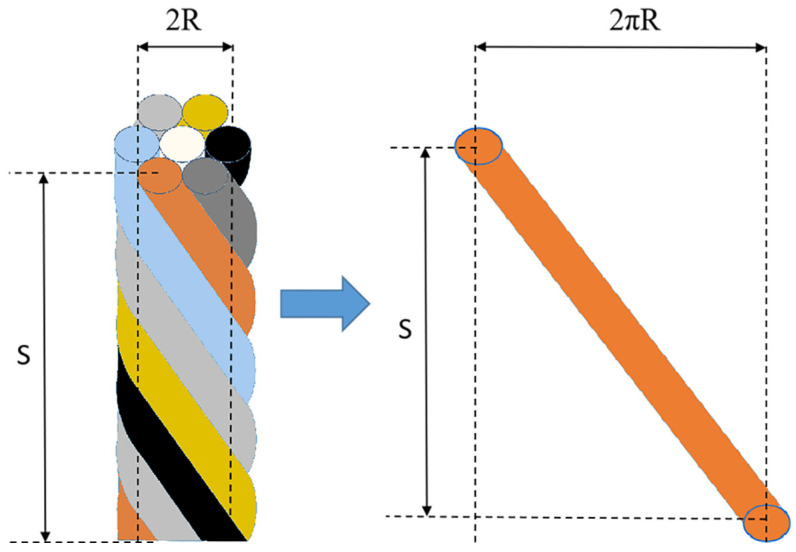
Cable wire architecture.

A core-sheath wire architecture was designed, consisting of a central 0.8 mm Al-Si wire surrounded by *n* = 4 peripheral 0.3 mm wires (Ti, Cu, Ni, Cr) helically wound at a specified radius R and pitch S (Figure 4). Unwinding a single pitch results in a helical structure with a developed length given by the following expression:

(22) $$L=\sqrt{{\left(2\pi R\right)}^{2}+S^{2}}$$

Hence the length ratio of sheath to core wires is:

(23) $$\frac{L}{S}=\frac{\sqrt{{\left(2\pi R\right)}^{2}+S^{2}}}{S}$$

and the ratio of the helical equivalent cross-sectional area s_1_ to the planar area s_2_ is:

(24) $$\frac{s_{1}}{s_{2}}=\frac{\sqrt{{\left(2\pi R\right)}^{2}+S^{2}}}{S}$$

Because the entire wire melts and alloys with the substrate, the mass feed rate per unit length of clad-track is:

(25) $$M=\frac{V_{f}}{V_{s}}\cdot S_{f}\cdot \rho_{f}$$

where V_f_ is the wire-feed speed, V_s_ the laser-scan speed, S_f_ the effective wire cross-section, and ρ_f_ the average wire density. For the cable-wire:

(26) $$S_{f}={{\pi r}_{\mathrm{in}}}^{2}+{{n\pi r}_{\mathrm{out}}}^{2}\cdot \frac{s_{1}}{s_{2}}$$

with r_in_ the core radius and r_out_ the peripheral-wire radius. Substituting (24) and (26) into (25) gives:

(27) $$M=\frac{V_{f}}{V_{s}}\cdot \left({{\pi r}_{\mathrm{in}}}^{2}\rho_{\mathrm{in}}+{{n\pi r}_{\mathrm{out}}}^{2}\rho_{\mathrm{out}}\cdot \frac{\sqrt{{\left(2\pi R\right)}^{2}+S^{2}}}{S}\right)$$

The cross-sectional area of the clad track is therefore:

(28) $$S_{m}=\frac{M}{\rho }=\frac{V_{f}}{V_{s}}\cdot \left({{\pi r}_{\mathrm{in}}}^{2}+{{n\pi r}_{\mathrm{out}}}^{2}\cdot \frac{\sqrt{{\left(2\pi R\right)}^{2}+S^{2}}}{S}\right)$$

**Figure 5:**
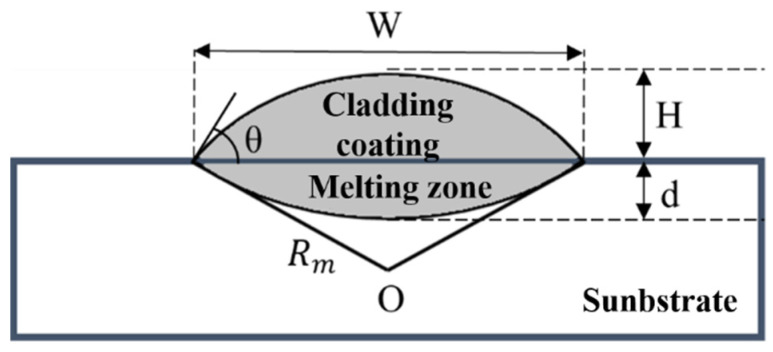
Typical clad-layer cross-section.

Figure 5 illustrates the clad cross-section, characterized by width W, height H, contact angle θ, penetration depth d, arc radius R_m_, and area S_m_. In practical terms, the cross-sectional profile can be closely approximated by a circular arc, the center O of which is determined by θ. For θ < 90° (which is the case for the current materials and process parameters), the center is located within the substrate. The geometric relationships lead to the following expressions:

(29) $$S_{m}=\arcsin\frac{W}{2R_{m}}\cdot {R_{m}}^{2}-\frac{W}{2}\sqrt{{R_{m}}^{2}-{\left(\frac{W}{2}\right)}^{2}}$$

(30) $$H=R_{m}-\sqrt{{R_{m}}^{2}-{\left(\frac{W}{2}\right)}^{2}}$$

Combining (28) and (29) gives the governing identity for cable-wire cladding under the present conditions:

(31) $$\frac{V_{f}}{V_{s}}\cdot \left({{\pi r}_{\mathrm{in}}}^{2}+{{n\pi r}_{\mathrm{out}}}^{2}\cdot \frac{\sqrt{{\left(2\pi R\right)}^{2}+S^{2}}}{S}\right)=\arcsin\frac{W}{2R_{m}}\cdot {R_{m}}^{2}-\frac{W}{2}\sqrt{{R_{m}}^{2}-{\left(\frac{W}{2}\right)}^{2}}$$

#### 3.6.2. Mathematical Model for Clad-Track Width W

Several factors influence clad quality; however, the clad-track width is predominantly determined by the incident laser heat flux. Steen et al. [43] established a correlation between clad-track width, laser-spot radius, and scanning speed, proposing the following relationship:

(32) $$W=2r_{las}\left(1-aV_{s}\right)$$

where a is an empirical constant. Yet this expression neglects the influence of laser power. For contact angles θ < 90°, the clad-track width corresponds to the substrate-melt width and is controlled by the heat-affected zone and volumetric heat input. Therefore, width is expected to increase with laser power and decrease with scanning speed.

To incorporate laser power, the original correlation is modified by introducing the line-energy density:

(33) $$b=\frac{P_{las}}{V_{s}}$$

which represents the heat input per unit clad-track length. The revised empirical relation becomes:

(34) $$W=2r_{las}\left(1+ab\right)$$

Table 1 lists the experimentally measured clad-track widths obtained under various process parameters. Solving Equation (34) with these data and averaging yields: a = 3.72 × 10^−8^ m/W·s.

Consequently, the final predictive equation for clad-track width is:

(35) $$W=2r_{\mathrm{las}}\left(1+3.72\times 10^{-8}b\right)$$

## 4. Results and Discussions

### 4.1. Molten Pool Behavior Analysis

**Figure 6:**
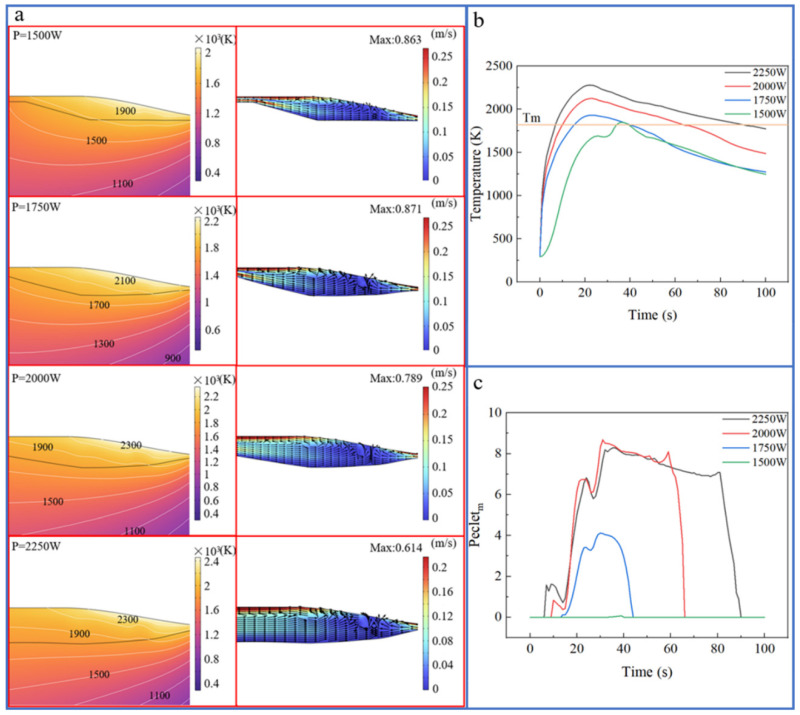
(**a**) Temperature fields and velocity fields in the longitudinal section of the molten pool at different power levels (t = 10 s); (**b**) temperature histories at a fixed monitoring point on the substrate surface during the entire cladding process under different power levels; (**c**) Peclet number at the same monitoring point at different power levels.

Figure 6a presents the temperature and velocity field distributions of the molten pool longitudinal section for cable wires with a pitch of 4 mm under different laser power conditions. As laser power increases, the peak temperature rises monotonically, and isotherms become denser, indicating a steeper thermal gradient. In contrast, the peak molten pool velocity exhibits a non-monotonic response: it increases from 1500 W, reaches a maximum of 0.871 m/s at 1750 W, and subsequently decreases to 0.614 m/s at 2250 W. This behavior reflects a transition in the dominant energy–momentum coupling mechanism. Between 1500 W and 1750 W, intensified Marangoni shear and reduced melt viscosity dominate, accelerating molten pool flow. Beyond 1750 W, the characteristic molten pool length expands rapidly; the prolonged transverse flow path enhances viscous dissipation, ultimately reversing the velocity trend.

The expanded characteristic length exerts a dual effect: it prolongs the residence time of molten metal, thereby extending the “time window” available for elemental diffusion, but simultaneously increases the viscous flow path length, reducing the characteristic velocity. Consequently, simply increasing laser power does not guarantee improved metallurgical homogenization; excessive power may even deteriorate mixing efficiency due to velocity decay. To quantify this trade-off, the molten pool lifetime (duration above the liquidus temperature T_m_) at a fixed substrate surface monitoring point was extracted from the computed thermal histories (Figure 6b), with corresponding characteristic lengths summarized in Table 2. As power increases from 1500 W to 2250 W, the characteristic length expands from 1.2 mm to 16.4 mm. At 1500 W, the short characteristic length (1.2 mm) and limited residence time are insufficient to homogenize the inherent radial compositional gradient of the cable wire, leading to poor coating formation.

To evaluate the competition between characteristic length and convective mixing, the molten pool Péclet number (Peclet_m_) is introduced [22], defined as Peclet_m_ = V_s_·L/D, where V_s_ is the characteristic velocity, L is the characteristic length, and D is the mass diffusion coefficient. A Peclet_m_ > 1 indicates convection-dominated mixing, with larger values corresponding to higher compositional uniformity. Figure 6c illustrates the temporal evolution of Peclet_m_ at the monitoring point. At 1500 W, Peclet_m_ reaches a maximum of only 0.07, indicating extremely weak convective mixing. At 1750 W, the peak Peclet_m_ ≈ 4 exceeds the threshold for conventional cladding but remains insufficient to eliminate the cable wire’s intrinsic radial segregation. At 2000 W, the peak Peclet_m_ increases to 8, ensuring vigorous convective mixing and significantly improved elemental homogeneity. Further increasing power to 2250 W slightly reduces the peak Peclet_m_ while introducing risks of grain coarsening, residual stress concentration, and elemental loss. Integrating the Péclet criterion with process stability considerations, the theoretical optimal laser power is identified as 2000 W, where the characteristic length and convective velocity achieve a balanced state, ensuring complete homogenization of the cable-wire’s radial compositional gradient and yielding a uniform, high-quality cladding layer. Experimental validation confirms that the cladding performance is optimal at 2000 W [12].

### 4.2. Solidification Analysis

**Figure 7:**
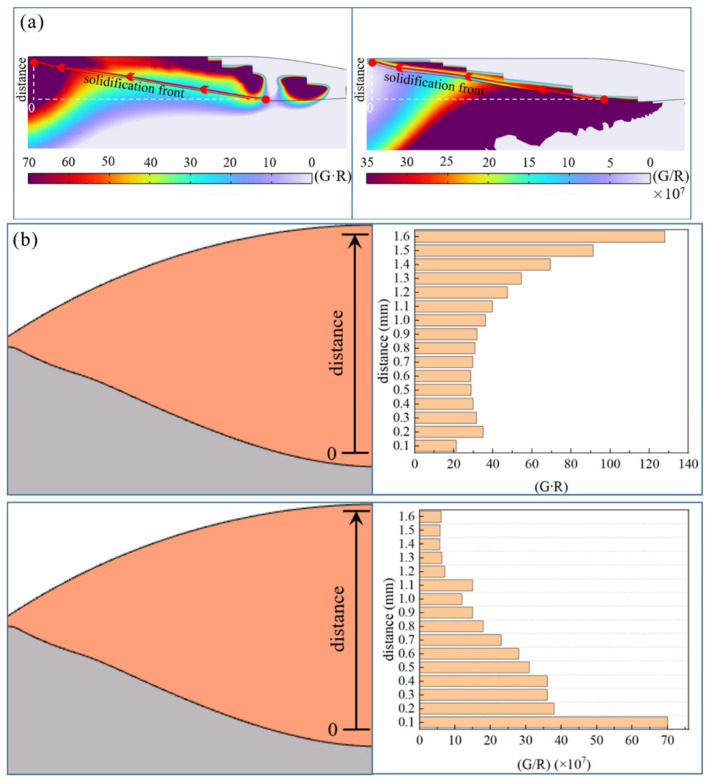
(**a**) Distribution of nucleation parameters (G·R) and morphological parameters (G/R) at the solidification front of the molten pool (P = 2000 W); (**b**) the grain nucleation parameters (G·R) and morphology parameters (G/R) of the coating cross-section vary with distance distribution (P = 2000 W).

**Figure 8:**
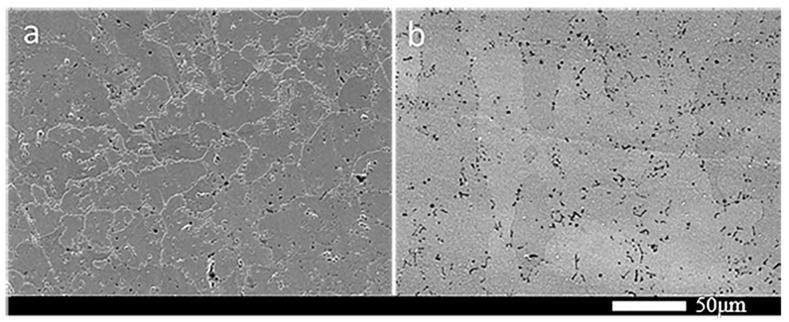
(**a**) Upper part of cladding coating; (**b**) lower part of cladding coating.

The molten pool behavior directly governs the subsequent solidification process, as the thermal gradient, flow velocity, and compositional distribution within the pool determine the nucleation and growth of grains during solidification. Figure 7a,b quantifies the solidification trajectory using the nucleation parameter (G·R) and the morphology parameter (G/R), where G is the temperature gradient and R is the solidification rate. From the molten pool base to the free surface, the monotonically increasing G·R amplifies constitutional undercooling, facilitating grain refinement. Conversely, the decreased G/R at the top of the pool promotes high solidification velocity, favoring equiaxed grain formation, while the elevated G/R at the bottom induces epitaxial growth of columnar grains along the steepest temperature gradient. The computed gradient-driven columnar-to-equiaxed transition (CET) is consistent with our previous experimental microstructural observations [12], as shown in Figure 8, validating the reliability of the solidification module in the proposed multi-physics model.

### 4.3. Stress Analysis

**Figure 9:**
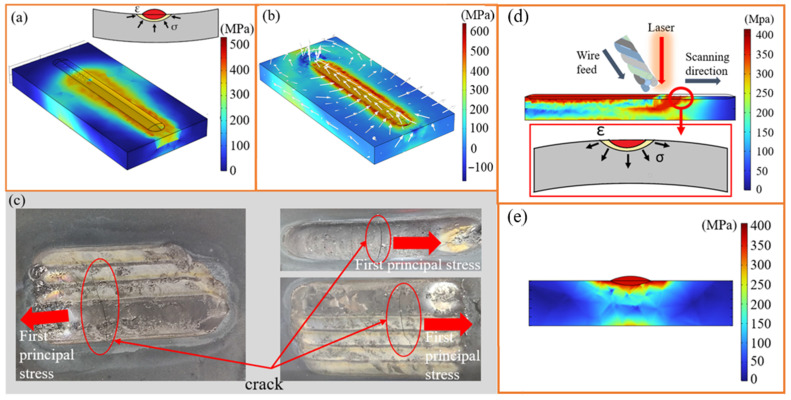
(**a**) Simulation results of the overlay layer stress (P = 2000 W); (**b**) directional diagram of the principal stress; (**c**) condition of the test crack; (**d**) stress distribution diagram of the molten layer’s longitudinal section; (**e**) residual stress distribution diagram of the molten layer’s cross section.

Residual stress is a critical factor affecting the mechanical integrity and service life of laser-cladded coatings. Figure 9a illustrates the stress distribution in the post-laser-cladding region, where significant residual stresses are observed in both the cladding zone and its surrounding areas. During the laser cladding process, non-uniform thermal expansion and contraction lead to the development of complex multi-axial stress states, including tensile, compressive, and shear stresses. A thorough investigation into the spatial distribution and interaction mechanisms of these stresses is therefore essential to understand their contribution to crack initiation and propagation. As shown in Figure 9b, stress magnitudes exhibit directional dependence: the maximum stress occurs along the laser scanning direction, followed by the stress perpendicular to the scanning path, while the minimum stress is observed in the direction normal to the substrate surface. This anisotropic stress distribution renders the clad layer susceptible to transverse through-cracks, consistent with the experimental findings presented in Figure 9c. To address this issue, it is recommended to optimize processing and material parameters to minimize residual stress formation. Furthermore, modifying the scanning strategy—such as adopting a spiral scanning path instead of a linear reciprocating pattern in area cladding—can effectively reduce residual stress accumulation. Figure 9d shows that rapid localized heating ahead of the molten pool generates compressive stresses; the thermal expansion of newly deposited material is elastically constrained by the colder substrate, forming a through-thickness compressive stress zone. Upon cooling (Figure 9e), the mismatch in thermal expansion coefficient and thermal conductivity between the cladding alloy and the substrate—amplified by the steep temperature gradient normal to the interface—leads to the concentration of tensile residual stress along the fusion boundary. This stress localization constitutes a dominant driving force for interfacial crack initiation and propagation. Therefore, the thermophysical compatibility between the alloy and the substrate must be considered a primary design variable to mitigate cracking susceptibility during cable-wire laser cladding.

**Figure 10:**
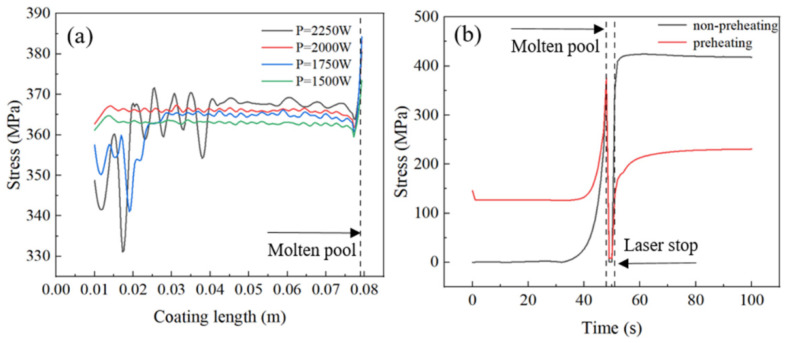
(**a**) Stress diagrams of different power cladding layers; (**b**) stress diagrams after cladding stops under different heat treatments (P = 2000 W).

Figure 10a demonstrates that the steady-state longitudinal stress at the cladding center increases monotonically with laser power, saturating at 365 MPa when P = 2000 W. At the deposition front, however, excessive power amplifies the spatial stress gradient, increasing the driving force for crack initiation. Thus, a reduced initial power followed by rapid ramp-up is recommended to minimize transient stress concentrations. Figure 10b shows that preheating the substrate to 400 °C reduces the final residual stress from 400 MPa to 200 MPa by attenuating the cooling temperature gradient, thereby suppressing solidification cracking without the need for additional post-processing. This provides a practical and efficient approach to stress control in cable-wire laser cladding.

### 4.4. Element Distribution Analysis

**Figure 11:**
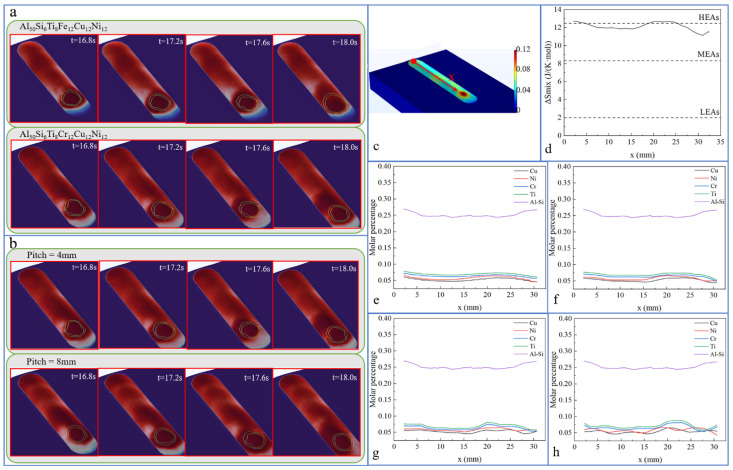
(**a**) Element concentration map of the coating layer of different component filaments (P = 2000 W, S = 4 mm); (**b**) element concentration map of the cable filament cladding layer for different pitch values (pitch S = 4 mm and S = 8 mm); (**c**) data points; (**d**) element mixing entropy; molar fraction of coating elements under different cable-wire pitch S (**e**) S = 2 mm; (**f**) S = 4 mm; (**g**) S = 6 mm; (**h**) S = 8 mm.

Elemental homogeneity is a key indicator of HEA coating quality, as it directly influences the formation of single-phase solid solutions and the resultant properties. The cable-wire pitch and composition significantly affect the elemental diffusion process within the molten pool, thereby governing the compositional uniformity of the coating. Figure 11a presents the Ti element mass transfer concentration distributions during cladding using Al_50_Si_6_Ti_8_Fe_12_Cu_12_Ni_12_ and Al_50_Si_6_Ti_8_Cr_12_Cu_12_Ni_12_ cable wires. Comparison of the two compositions reveals more pronounced elemental diffusion in Al_50_Si_6_Ti_8_Cr_12_Cu_12_Ni_12_, with its mass transfer concentration diagram exhibiting a distinct left–right oscillation pattern consistent with the helical structure of the outer cable wire strands. In contrast, Al_50_Si_6_Ti_8_Fe_12_Cu_12_Ni_12_ shows large-area elemental aggregation, with less evident diffusion and poor elemental homogeneity.

This difference is attributed to the high surface activity of Cr, which exhibits lower atomic adsorption energy on the melt surface. As a result, Cr modifies the microstructure and energy state of the melt surface, weakens intermolecular forces, and reduces melt viscosity. JMatPro simulation results indicate that the addition of 12% Cr reduces melt viscosity by approximately 15%, enhancing melt fluidity and mixing efficiency. These findings demonstrate that rational cable-wire compositional design can significantly improve elemental homogeneity in the cladding layer. For example, increasing the proportion of low-melting-point elements promotes melt flow and mixing, while the addition of appropriate surface-active elements modifies the melt surface tension distribution, thereby regulating flow behavior and elemental distribution.

Figure 11b shows that reducing the cable-wire pitch (S) at a constant wire-feed speed increases the effective torsional frequency of the strands within the molten pool and shortens the residence time of individual peripheral strands at a fixed spatial node. The intensified material exchange suppresses local solute accumulation; in contrast, a pronounced segregation pattern emerges when S = 8 mm. Thus, maintaining S ≤ 4 mm is critical for ensuring lateral compositional uniformity. Figure 11d further reveals that solute-depleted regions exhibit a significant decrease in configurational entropy (ΔS_mix_), while the average ΔS_mix_ of the coating approaches the HEA threshold. This indicates a strong thermodynamic driving force for single-phase solid-solution formation and concurrently mitigates the nucleation of harmful intermetallic compounds.

Figure 11e–h presents the elemental mole fractions in coatings fabricated using cable-wires with different pitches. The compositional homogeneity of the outer strands decreases significantly with increasing pitch. When S = 2 mm or 4 mm, the elemental distribution remains relatively uniform. However, when S = 6 mm, the elemental mole fractions exhibit periodic fluctuations corresponding to the cable wire’s self-rotational structure. At S = 8 mm, severe elemental segregation occurs. The substantial variation in elemental mole fractions for larger pitches indicates that increasing the pitch significantly degrades elemental distribution uniformity. Therefore, for this type of composite cable wire, the pitch should be controlled within ≤4 mm to ensure optimal coating elemental uniformity.

## 5. Conclusions

A fully coupled multi-physics model was developed to simulate the laser cladding process of Al_50_Si_6_Ti_8_Cr_12_Cu_12_Ni_12_ cable wires. The twisted multi-strand feedstock was explicitly modeled, and its self-rotation and associated mass transfer within the molten pool were captured using an Arbitrary Lagrangian-Eulerian (ALE) moving-mesh solver coupled with a Level Set interface tracker. Systematic validation against in situ process diagnostics revealed the quantitative effects of operational and compositional variables on the thermal field, flow pattern, cladding topography, residual stress, solute distribution, and microstructural evolution. Mechanistic analyses clarified the molten pool mixing kinetics and crack-driving stress states, establishing material–process–performance correlations that enable a priori prediction of compositional uniformity. The key conclusions are as follows:

(1) A critical molten pool Péclet number (Peclet_m_ ≥ 8), achieved at a laser power of 2000 W, marks the transition from diffusion-dominated to convection-dominated mixing, eliminating the intrinsic radial segregation of the cable wire. Powers exceeding 2250 W reverse this trend due to viscous dissipation and increase defect susceptibility.
(2) The columnar-to-equiaxed transition (CET) is governed by the gradients of G·R and G/R. The predicted grain morphology is in exact agreement with previous experimental micrographs, validating the solidification module of the model.
(3) Tensile residual stress concentrates at the cladding–substrate interface due to thermophysical property mismatch. Preheating the substrate to 400 °C halves the stress amplitude and suppresses crack initiation without the need for additional post-processing.
(4) The surface-active element Cr reduces melt viscosity by approximately 15%, enhancing momentum and mass transfer. Conversely, increasing the cable-wire pitch lengthens elemental residence loops and induces periodic segregation. Thus, a pitch of S ≤ 4 mm is required to maintain lateral homogeneity and retain the coating’s configurational entropy within the HEA regime.
(5) Integrating alloy chemistry, wire architecture, and laser parameters into a unified Péclet–stress–entropy framework provides a priori design guideline for fabricating crack-free, compositionally uniform HEA coatings via cable-wire laser deposition.

However, several limitations remain that warrant future investigation. First, the current stress field simulation relies on steady-state mechanical parameters extracted from JMatPro, which inherently limits the model’s ability to account for transient microstructural evolution and composition-dependent property variations during solidification. Incorporating fully coupled transient mass transfer effects into the mechanical analysis represents a critical next step toward achieving higher-fidelity stress field predictions. Additionally, to further advance the theoretical framework for lightweight high-entropy alloys, future studies should elucidate the underlying phase transformation mechanisms and elemental synergistic effects—particularly the dynamic interactions among multiple elements during rapid solidification. Addressing these aspects will enable more comprehensive process–microstructure–property relationships in laser-based additive manufacturing of advanced alloys.

## Figures and Tables

**Table 1:** Clad-layer width under different process parameters.

No.	Laser Power P_las_ (W)	Scanning Speed V_s_ (mm/s)	Measured Width W (mm)
1	1500	0.2	7.3
2	1500	0.2	7.5
3	1750	0.2	7.9
4	1750	0.2	7.7
5	2000	0.2	8.5
6	2000	0.2	9.0
7	2250	0.2	9.0
8	2250	0.2	9.2
9	1825	0.2	8.0
10	1900	0.2	9.0
11	1500	0.1	8.3
12	1750	0.1	9.1
13	1825	0.1	9.1
14	2000	0.1	9.8
15	2250	0.1	10.1
16	1825	0.3	7.7

**Table 2:** Melt-pool characteristic lengths at the same monitoring point at different power levels.

Laser Power (W)	Melt-pool Characteristic Length (mm)
1500	1.2
1750	5.2
2000	11.0
2250	16.4

## Data Availability

The original contributions presented in this study are included in the article. Further inquiries can be directed to the corresponding authors.

## References

[1] Hur S.M., Choi K.H., Lee J.K. (2001). Determination of fabricating orientation and packing in SLS process. J. Mater. Process Technol..

[2] Lu Z.L., Li D.C., Tong Z.Q., Lu Q.P., Traore M.M., Zhang A.F., Lu B.H. (2011). Investigation into the direct laser forming process of steam turbine blade. Opt. Laser Eng..

[3] Zhu G.X., Zhang A.F., Li D.C. (2009). Optimization of laser cladding process parameters based on Nd:YAG laser. Appl. Laser.

[4] Zhang M. (2013). Research on the Characteristics of Laser Additive Manufacturing of Titanium Alloy Using Powder Feeding and Wire Feeding Methods. Master’s Thesis.

[5] Chen J.X., Li T., Chen Y., Wang J., Wang Y.D., Lu Z.P. (2023). Ultra-strong heavy-drawn eutectic high entropy alloy wire. Acta Mater..

[6] Capello E., Colombo D., Previtali B. (2005). Repairing of sintered tools using laser cladding by wire. J. Mater. Process Technol..

[7] Zhong M.L., Liu W.J. (2008). Dominant fields and hotspots in international laser material processing research. Chin. J. Lasers.

[8] Zhang D.Y., Wang R.Z., Zhao J.Z., Zuo T. (2010). Recent advances in laser direct manufacturing of metal parts. Chin. J. Lasers.

[9] Kim J.D., Peng Y. (2000). Plunging method for Nd:YAG laser cladding with wire feeding. Opt. Laser Eng..

[10] Ding D.H., Pan Z.X., Cuiuri D., Li H.J., Xu J., Norrish J. (2014). A tool-path generation strategy for wire and arc additive manufacturing. Int. J. Adv. Manuf. Technol..

[11] Ding D.H., Pan Z.X., Cuiuri D., Li H.J., Xu J., Norrish J. (2015). A practical path planning methodology for wire and arc additive manufacturing of thin-walled structures. Robot Comput Integr. Manuf..

[12] Liu W., Jiang K., Yan B., Liang H.Y., Li Y., Wang Y. (2024). Cable-wire-fed laser cladding of high-aluminum lightweight high-entropy alloys. Mater. Today Commun..

[13] Wang W., Zheng Y., Cai Z., Zheng W., Zhang C., Wang Y., Zhao Z., Feng D., Ma Y., Yang J. (2024). High-entropy alloy laser cladding with cable-type welding wire: Experimental study and first-principles calculations. Metals.

[14] Zhang J., Li M., Chen Z., Wu D., Zheng C., Huang K., Yi X. (2025). Numerical simulation and experimental research on the effects of substrate preheated on the cracks, microstructure, and properties of laser cladding WC-Ni60AA coatings. Mater. Today Commun..

[15] Jiang Y.C., Cheng Y., Zhang X., Yang J., Yang X., Cheng Z.Q. (2020). Simulation and experimental investigations on the effect of Marangoni convection on thermal field during laser cladding process. Optik.

[16] Hu S.P., Sun L.F., Gao Y.C., Zhang C., Yu T.B. (2025). Macroscopic temperature field modeling and simulation of nickel-based cladding layers in laser cladding. Appl. Sci..

[17] Dao M.Y., Lou J. (2021). Simulations of laser assisted additive manufacturing by smoothed particle hydrodynamics. Comput Methods Appl. Mech. Eng..

[18] Gan Z.T., Yu G., He X.L., Li S.X. (2017). Surface-active element transport and its effect on liquid metal flow in laser-assisted additive manufacturing. Int. Commun. Heat. Mass. Transf..

[19] Wang L.P., Zhang D.C., Chen C.Z., Fu H., Sun X.M. (2022). Multi-physics field coupling and microstructure numerical simulation of laser cladding for engine crankshaft based on CA-FE method and experimental study. Surf. Coat. Technol..

[20] Li C., Xu Y., Jia T.H., Zhao J.J., Han X. (2022). Numerical simulation research on multifield coupling evolution mechanism of IN625 laser cladding on nodular cast iron. Int. J. Adv. Manuf. Technol..

[21] Batchelor G.K. (2000). An Introduction to Fluid Dynamics.

[22] Li G., Wang Z., Yao L., Zhang K.Y., Liang Y.J. (2023). Component mixing in laser cladding processes: From single-track to single-layer multi-track and multi-layer multi-track. Surf. Coat. Technol..

[23] Cho J.H., Farson D.F., Milewski J.O., Hollis K.J. (2009). Weld pool flows during initial stages of keyhole formation in laser welding. J. Phys. D. Appl. Phys..

[24] Sun Z., Guo W., Li L. (2020). Numerical modelling of heat transfer, mass transport and microstructure formation in a high deposition rate laser directed energy deposition process. Addit. Manuf..

[25] Tang C., Tan J.L., Wong C.H. (2018). A numerical investigation on the physical mechanisms of single track defects in selective laser melting. Int. J. Heat. Mass. Transf..

[26] Gong Y., Wang L.F., Zhu G.X., Li D.C. (2018). Numerical simulation study of residual stress in laser cladding layers of 316L stainless steel. Appl. Laser.

[27] Fu W., Fang H.Y., Bai X.B., Zhang X., Li H. (2019). The impact of process path on residual stress in multi-layer multi-pass laser cladding. J. Weld..

[28] Sun S.T., Fu H.G., Chen S.Y., Ping X.L., Wang K.M., Guo X.Y., Lin J.L., Lei Y.P. (2019). A numerical-experimental investigation of heat distribution, stress field and crack susceptibility in Ni60A coatings. Opt. Laser Technol..

[29] Yu T.B., Qiao R.Z., Han J.B., Liu W., Yang J. (2020). Numerical simulation of the residual stress field in laser cladding on tilted substrates. Hot Work Technol..

[30] Jiang Q.Y. (2011). Simulation analysis of stress fields in multi-pass laser cladding. Hot Work Technol..

[31] Cai G.C., Yin Y., He C.Y., Zhang J., Wang Y. (2019). Stress analysis of the quenching process of 2A12 aluminum alloy thick plate. Light Alloy Fabr. Technol..

[32] Wang Y.B., Jin G.Y., Zhang W. (2016). Temperature and thermal stress analysis of aluminum alloy sheets under long-pulse laser action. Chin. J. Laser.

[33] Tu S.J., Zheng Y., Wang H., Ma N., Wang Z.M. (2019). Comparison of welding deformation and residual stress between laser welding and inert gas shielded welding of SUS304 stainless steel thin plates. Laser J..

[34] Chen Z.Y., Zhu E.C., Pan J.L. (2011). Numerical simulation of the mechanical properties of wood under complex stress states. Chin. J. Comput Mech..

[35] Wang Z.Y., Chen Y., Wang S.G. (2018). Progress in numerical simulation analysis of high-energy beam welding of TiAl alloys. Weld. Technol..

[36] Chen J., Tan H., Yang H.O., Lin X., Huang W.D. (2007). Evolution of molten pool shape in the process of laser rapid forming. Chin. J. Lasers.

[37] Lalas C., Tsirbas K., Salonitis K., Chryssolouris G. (2007). An analytical model of the laser clad geometry. Int. J. Adv. Manuf. Technol..

[38] Tabernero I., Lamikiz A., Martinez S., Ukar E., de Lacalle L.N.L. (2012). Geometric modelling of added layers by coaxial laser cladding. Phys. Procedia.

[39] Toyserkani E., Khajepour A., Corbin S. (2003). Three-dimensional finite element modeling of laser cladding by powder injection: Effects of powder feedrate and travel speed on the process. J. Laser Appl..

[40] Toyserkani E., Khajepour A., Corbin S. (2004). 3-D finite element modeling of laser cladding by powder injection: Effects of laser pulse shaping on the process. Opt. Laser Eng..

[41] Davim J.P., Oliveira C., Cardoso A. (2008). Predicting the geometric form of clad in laser cladding by powder using multiple regression analysis. Mater. Des..

[42] Onwubolu G.C., Davim J.P., Oliveira C., Cardoso A. (2007). Prediction of clad angle in laser cladding by powder using response surface methodology and scatter search. Opt. Laser Technol..

[43] Steen W.M., Weerasinghe V.M., Monson P. (1986). Some aspects of the formation of laser clad tracks. High Power Lasers and Their Industrial Applications.

